# Ginaton reduces M1-polarized macrophages in hypertensive cardiac remodeling *via* NF-κB signaling

**DOI:** 10.3389/fphar.2023.1104871

**Published:** 2023-03-13

**Authors:** Jie Wang, Enze Cai, Xiangbo An, Junjie Wang

**Affiliations:** ^1^ Department of Cardiology, Affiliated Zhongshan Hospital of Dalian University, Dalian, China; ^2^ Department of Cardiology, the Fifth People’s Hospital of Dalian, Dalian, China; ^3^ Department of Interventional Therapy, First Affiliated Hospital of Dalian Medical University, Dalian, China; ^4^ Department of Cardiology, First Affiliated Hospital of Dalian Medical University, Dalian, China

**Keywords:** ginaton, inflammation, hypertension, cardiac remodeling, macrophage polarization

## Abstract

**Introduction:** Macrophages play a critical role in cardiac remodeling, and dysregulated macrophage polarization between the proinflammatory M1 and anti-inflammatory M2 phenotypes promotes excessive inflammation and cardiac damage. Ginaton is a natural extract extracted from *Ginkgo biloba*. Because of its anti-inflammatory properties, it has long been used to treat a variety of diseases. However, the role of Ginaton in modulating the diverse macrophage functional phenotypes brought on by Ang II-induced hypertension and cardiac remodeling is unknown.

**Methods:** In the present study, we fed C57BL/6J mice in the age of eight weeks with Ginaton (300 mg/kg/day) or PBS control, and then injected Ang II (1000 ng/kg/min) or saline for 14 days to investigate the specific efficacy of Ginaton. Systolic blood pressure was recorded, cardiac function was detected by echocardiography, and pathological changes in cardiac tissue were assessed by histological staining. Different functional phenotypes of the macrophages were assessed by immunostaining. The mRNA expression of genes was assessed by qPCR analysis. Protein levels were detected by immunoblotting.

**Results:** Our results showed that Ang II infusion significantly enhanced the activation and infiltration of macrophages with hypertension, cardiac insufficiency, myocardial hypertrophy, fibrosis and M1 phenotype macrophages compared with the saline group. Instead, Ginaton attenuated these effects. In addition, *in vitro* experiments showed that Ginaton inhibited Ang II-induced activation, adhesion and migration of M1 phenotype macrophages.

**Conclusion:** Our study showed that Ginaton treatment inhibits Ang II-induced M1 phenotype macrophage activation, macrophage adhesion, and mitigation, as well as the inflammatory response leading to impaired and dysfunctional hypertension and cardiac remodeling. Gianton may be a powerful treatment for heart disease.

## Introduction

Cardiovascular disease is a major public health concern worldwide (Benjamin et al., 2019). Hypertension is the leading cause of cardiovascular disease, affecting approximately 1 billion people globally (Mouton et al., 2020). The prevalence of hypertension is increasing by more than 10% every year (Houston et al., 2011). Recent studies have shown that chronic high blood pressure can lead to cardiac hypertrophy, inflammation, fibrosis, and, ultimately, heart failure (Peet et al., 2020). Therefore, therapeutic strategies aimed at alleviating hypertension and preventing myocardial hypertrophy, inflammation, and fibrosis may help prevent hypertension-induced cardiac insufficiency or heart failure (Berthiaume et al., 2012; Dodd et al., 2012; Cavalera et al., 2014). During the development of hypertension, chronic pressure overload is closely associated with myocardial hypertrophy and inflammation, increasing the risk of heart failure and sudden death (DeBerge et al., 2019). Angiotensin II (Ang II) is a bioactive octapeptide derived from the renin–angiotensin–aldosterone system. Elevated levels of Ang II and its receptor type 1 angiotensinogen (AT1) promote cardiac remodeling and play an important role in the occurrence and development of hypertension (Yin et al., 2022). In addition to the known hemodynamic effects, Ang II also mediates cardiac remodeling through multiple signaling pathways, including protein kinase B/mammalian target of rapamycin (AKT/mTOR), mitogen-activated protein kinase (MAPK), nuclear factor-κB subunit (NF-κB), and transforming growth factor-β/mothers against decapentaplegic homolog (TGF-β/Smad) (Jin et al., 2015; Samak et al., 2016; Trial, et al., 2017; Mouton et al., 2018). Ang II stimulates hypertrophic remodeling by promoting proinflammatory cell infiltration into cardiac tissue. Recent studies have shown that Ang II stimulation can increase the adhesion of macrophages/monocytes to endothelial cells and subsequently migrate to cardiac tissue through endothelial cells, generate proinflammatory cytokines, activate multiple signaling pathways, and lead to cardiac remodeling (Peet et al., 2020). However, the roles of different functional phenotypes of macrophages in Ang II-induced hypertensive myocardial remodeling are unclear.

Macrophage monocytes are innate immune system pleiotropic cells that are critical for the initial inflammatory response and subsequent wound healing following injury in many tissues, including the heart (Labonte et al., 2014; Liu et al., 2014). Macrophages also play a central role in inflammation and host defense (Sica et al., 2012). Macrophages are monocyte-derived cells derived from proinflammatory (M1) and repair (M2) macrophages (Sica et al., 2007; Biswas et al., 2010). M1 phenotypic macrophages express many proinflammatory mediators, including tumor necrosis factor α (TNF-α), IL-1, IL-6, reactive nitrogen, and oxygen intermediates with potent microbicidal and tumoricidal activities, while M2 phenotypic expression molecules include resistin-like α (also known as FIZZ1), arginase 1 (Arg1), chitinase 3-like 3 (also known as Ym 1), IL-10, and Mrc1 (also known as CD206) (Gordon et al., 2010). These molecules are thought to be involved in parasite infection, tissue remodeling, and tumor progression (Mantovani et al., 2002). Recent studies have reported increased levels of proinflammatory cytokines, such as IL-6, secreted by M1 phenotypic macrophages in patients with unstable angina and myocardial infarction; moreover, high levels predict adverse outcomes (Hanna et al., 2020). *In vitro* studies have found that M1 phenotype macrophages can also induce smooth muscle cell proliferation and release vasoactive molecules, including NO, endothelin, and eicosanoids, which are important consequences of lipoprotein oxidation and cytotoxicity (Khallou-Laschet et al., 2010; Tsimikas et al., 2011; Ndisang et al., 2014). Early arteriosclerotic plaques are infiltrated by M2 phenotype macrophages; however, with plaque progression, M1 phenotype macrophages gradually increase and dominate (Khallou-Laschet et al., 2010). The present study investigated the role of different phenotypes of macrophages in Ang II-induced hypertensive myocardial remodeling to provide new opportunities for the treatment of cardiovascular diseases.

Ginaton is a natural product extracted from *Ginkgo biloba* that has been used to treat cardiovascular and cerebrovascular diseases for decades (Li et al., 2020). Ginaton has well-known anti-inflammatory, antioxidant, and antiapoptotic effects (Gevrek et al., 2018). Previous studies demonstrated that Ginaton can downregulate the TGF-β signaling pathway and effectively improve renal interstitial fibrosis and the inflammatory response (Liang et al., 2021). However, the role of Ginaton in mediating Ang II-induced cardiac remodeling remains unclear.

Herein, we investigated the role of macrophages of different phenotypes in Ang II-induced myocardial remodeling in hypertension and the potential protective effect of Ginaton on Ang II-induced cardiac remodeling, providing a new therapeutic target for cardiovascular diseases.

## Materials and methods

### Animal study

In this study, 8-week-old C57BL/6 mice purchased from Jackson Laboratory (Sacramento, CA) were treated as wild-type (WT). We treated the mice with Ang II (1,000 ng/kg/min) or saline for 14 days. The hypertensive myocardial remodeling models and controls were established using osmotic micropumps (Alzert Model 1002, DURECT, Cupertino, CA). Ginaton injection was purchased from Chi Sheng Chemical Cooperation (Taiwan, China). To investigate the potential protective effects of Ginaton, we administered Ginaton (300 mg/kg/day) to the mice intragastrically the day before surgery, at a dose according to that reported previously (Sun et al., 2015). Phosphate-buffered saline (PBS) treatment was used as a control. All animal experiments were carried out in accordance with the National Institutes of Health (NIH) Guidelines for the Care and Use of Laboratory Animals, which were approved by the Dalian Medical University Animal Committee.

### Measurement of systolic blood pressure and cardiac function

We used a tail-cuff device (BP-98, Softron, Japan) to record systolic blood pressure (SBP) and heart rate in all mice. We placed the mice on a retainer, completely exposing the tail, and then placed the tail-cuff device to measure their SBP and heart rate. Cardiac function was measured in each mouse using a 30 MHz probe (Vevo 1100 system, VisualSonics, Toronto, Canada). By measuring the echocardiography of each mouse, we recorded the end-diastolic and end-systolic left ventricular anterior wall (LVAW) thickness, left ventricular posterior wall (LVPW) thickness, ventricular inner diameter (ID), LV ejection fraction (EF%), and LV fractional shortening (FS%).

### Histopathology and immunofluorescence

Heart tissue was fixed in 4% POM for >24 h. Part of the heart tissue was paraffin-embedded, while the other part was OCT-embedded. Masson’s trichrome and hematoxylin and eosin (H&E) staining were performed on paraffin sections (4 μm). The OCT sections (8 μm) were treated with wheat germ agglutinin (WGA). Cardiac sections were stained with anti-α-SMA (ab124964, Abcam) by immunohistochemistry at 4°C overnight. The next day, after washing with PBS, the sections were incubated with the secondary antibody and DAB substrate. The color reaction was stopped with ddH2O, and the sections were incubated with hematoxylin. Frozen sections or cells were fixed with 4% POM for 15 min at room temperature and then incubated with anti-CD68 (ab283654, Abcam), anti-CD206 (ab300621, Abcam), or anti-iNOS (ab283655, Abcam) at 4°C overnight. The next day, after washing with PBS, the sections were incubated with fluorescently labeled antibodies at room temperature for 30 min and then with DAPI at room temperature for 3 min. Pictures of the sections were taken at ×100/×200 magnification on a fluorescence microscope (Olympus, BX53, Japan).

### RNA isolation and quantitative real-time PCR

Total RNA was extracted from fresh heart tissue or cells. cDNA (1 μg) was obtained using PrimeScript RT reagent (Takara, Japan). Quantitative real-time PCR was performed with an SYBR Green Premix Pro Taq HS qPCR Kit (AG11701). All mRNA levels were normalized to GAPDH by ΔΔCt analysis.

### Western blot analysis

Fresh heart tissue was added to RIPA lysis buffer to obtain total protein. Protein (25 μg) was subjected to SDS-PAGE and transferred to polyvinylidene fluoride membranes. The membranes were blocked with skim milk for 30 min at room temperature and then incubated with primary antibodies at 4°C overnight. The next day, after washing with TBST, the membranes were incubated with conjugated secondary antibodies (1:3000, CST) at room temperature for 1 h, and the bands were visualized by chemiluminescence. The main antibodies used in this study were: anti-TGF-β (ab215715, Abcam), anti-p-Smad2 (ab280888, Abcam), anti-Smad2 (ab33875, Abcam), anti-p-AKT (ab38449, Abcam), anti-AKT (orb11276, Biorbyt), anti-p-ERK1/2 (ab214036, Abcam), anti-ERK1/2 (ab184699, Abcam), anti-p-IKKα (ab138426, Abcam), anti-IKKα (ab32041, Abcam), anti-p-P65 (ab76302, Abcam), anti-P65 (ab16502, Abcam), and anti-GAPDH (ab8245, Abcam).

### Cell adhesion and migration analysis

Macrophages were obtained from the tibias and femurs of WT mice and cultured in 1640 medium (Meilunbio, MA0215). Human umbilical vein endothelial cells (HUVECs) were cultured in ECM. Proclones from macrophages to adherent HUVECs were distributed as previously described (Yin et al., 2022). We pretreated HUVECs with Ginaton (100 μg/ml) or PBS for 4 h and then with Ang II (100 nM) or saline for 24 h. Macrophages were labeled with PKH-26 fluorescent dye and added to HUVECs at a ratio of 10:1. After incubation for 1 h, the non-adherent cells were washed with PBS, while the adherent cells were photographed by microscopy.

HUVECs were cultured and treated as described in the adhesion experiments. Macrophages (5 × 10^4^) were added to the upper chamber of a 24-well Transwell plate (8 μm pore, Coning), and HUVEC-conditioned media were added to the lower chamber of the plate. After 24 h of incubation, the migrated cells were fixed, stained with DAPI, and observed by microscopy.

### Statistics

Data are presented as means ± SD. Statistical analysis was performed using GraphPad Prism software. For statistical comparison, a one-way ANOVA was used, followed by Dunnett’s multiple comparison tests with the control group. Student’s unpaired *t*-tests were used to compare the two groups. *p* <0.05 was considered statistically significant.

## Results

### Ginaton treatment alleviates hypertension and cardiac dysfunction caused by Ang II

**FIGURE 1:**
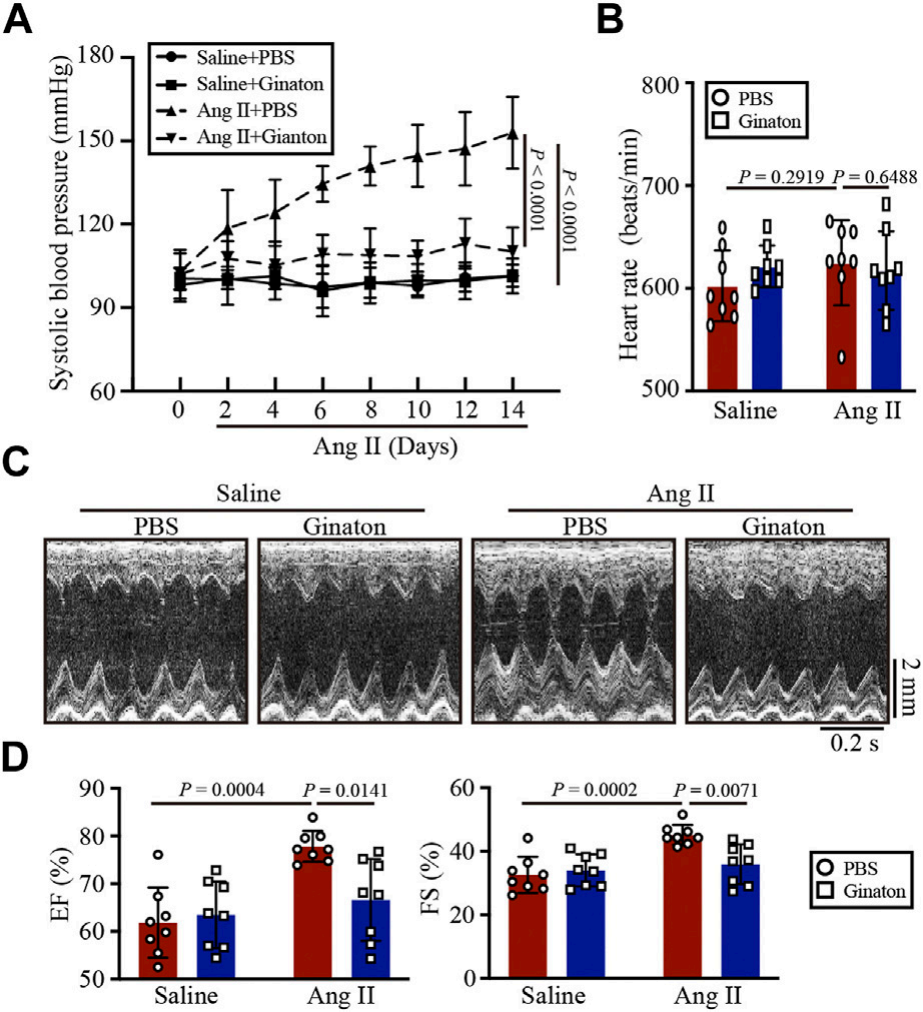
Treatment with Ginaton relieves Ang II-induced hypertension and cardiac dysfunction. **(A)** Ginaton (300 mg/kg/day) or PBS (internal control) was administered intragastrically to 8-week-old C57BL/6J mice. Mice were then infused with Ang II (1,000 ng/kg/min) or saline for 14 days. The average systolic blood pressures were recorded before and after Ang II infusion (n = 4). **(B)** Heart rates in each group (n = 8). **(C)** M-mode echocardiographs to assess left ventricular function (n = 8). **(D)** Ejection fractions (EF%) and fractional shortenings (FS%) (n = 8). The results are expressed as means ± SD; n, number of animals per group.

As shown in Figure 1A, WT mice were treated with Ginaton (300 mg/kg) and Ang II (1,000 ng/kg/min) for 14 days. PBS was used as the control. After 14 days of Ang II infusion, the SBP in the Ginaton-treated group was significantly lower than that in the PBS-treated group. However, after Ang II infusion, heart rates were similar in the Ginaton and PBS treatment groups (Figure 1B). Echocardiography showed enhanced cardiac function 14 days after Ang II infusion, with increased EF% and FS% in the PBS-treated group, while treatment with Ginaton effectively mitigated this response (Figures 1C, D, Supplementary Table S1).

### Ginaton treatment mitigates the Ang II-induced cardiac hypertrophic response

**FIGURE 2:**
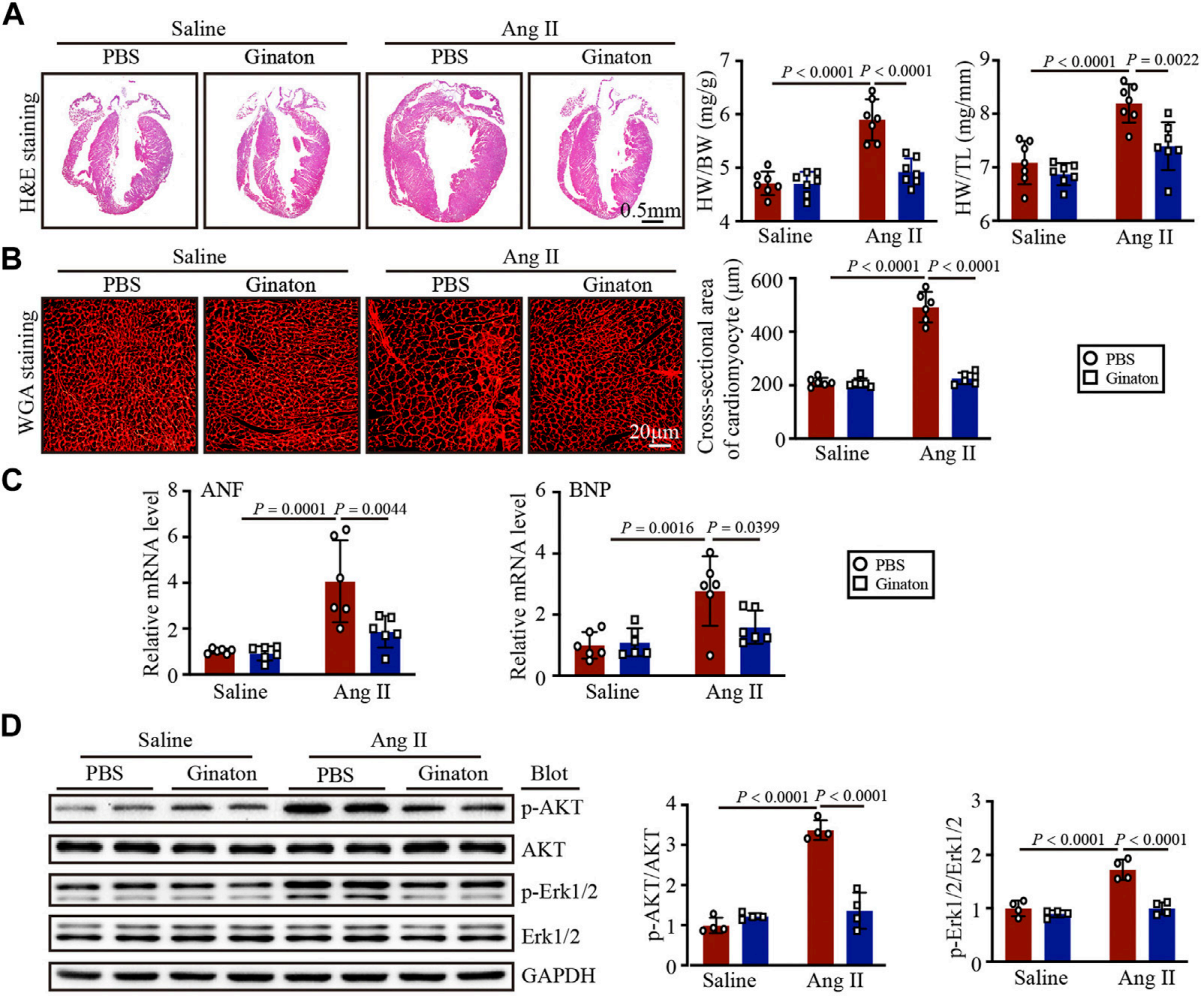
Ginaton administration rescues Ang II-induced cardiac hypertrophy. Ginaton (300 mg/kg/day) or PBS (internal control) was administered intragastrically to 8-week-old C57BL/6 J mice. Mice were then infused with Ang II (1,000 ng/kg/min) or saline for 14 days. **(A)** H&E staining of heart tissues in each group (left, n = 4, scale bar = 0.5 mm) and the ratios of heart weight to body weight (HW/BW) and HW to tibial length (HW/TL) (right, n = 8). **(B)** Heart sections stained with wheat germ agglutinin (WGA) (left, n = 6, scale bar = 20 μm) and quantification of myocyte cross-sectional areas (right, n = 6). **(C)** qPCR analyses of atrial natriuretic factor (ANF) and brain natriuretic peptide (BNP) mRNA levels (n = 6). **(D)** Western blot analyses of the expression of phosphorylated (p)-AKT, AKT, p-ERK1/2, ERK1/2, and GAPDH proteins in heart sections (left, n = 4) and the relative protein levels of each (right, n = 4). The results are expressed as means ± SD; n, number of animals per group.

Next, we determined the role of Ginaton in Ang II-induced cardiac hypertrophy. After 14 days of Ang II infusion, PBS-treated mice exhibited the characteristics of myocardial hypertrophy, including increased left ventricular thickness, heart weight/body weight (HW/BW), heart weight/tibial length (HW/TL), and increased cardiomyocyte cross-sectional area; however, these effects were mitigated by Ginaton (Figures 2A, B). Ginaton also reduced the expression of hypertrophy markers (ANF and BNP) after Ang II infusion (Figure 2C). The primer sequences are listed in Supplementary Table S2. As shown in Figure 2D, Ginaton downregulated the Ang II-induced activation of AKT and ERK1/2 expression. Therefore, Ginaton attenuated the Ang II-induced cardiac hypertrophy response.

### Ginaton treatment reduces Ang II-induced cardiac fibrosis

**FIGURE 3:**
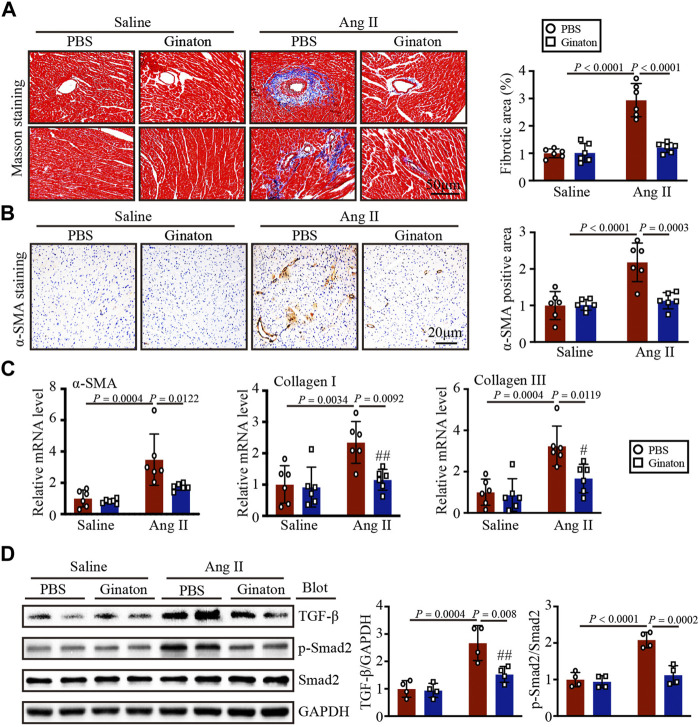
Ginaton reduces fibrosis and collagen deposition in Ang II-infused mice. Ginaton (300 mg/kg/day) or PBS (internal control) was administered intragastrically to 8-week-old C57BL/6J mice. Mice were then infused with Ang II (1,000 ng/kg/min) or saline for 14 days. **(A)** Masson’s trichrome staining of cardiac sections (left, n = 6, scale bar = 50 μm) and quantification of the areas of collagen deposition (right, n = 6). **(B)** Immunohistochemical staining of heart tissues with antibody against α-smooth muscle actin (α-SMA) (left, n = 6, scale bar = 20 μm); quantification of α-SMA^+^ areas (right, n = 6). **(C)** qPCR analyses of α-SMA, collagen I, and collagen III mRNA levels (n = 6). **(D)** Western blot analyses of the expression of TGF-β, p-Smad2, Smad2, and GAPDH proteins in heart sections (left, n = 4) and the relative protein levels of each (right, n = 4). The results are expressed as means ± SD; n, number of animals per group.

Masson’s trichrome staining to investigate the role of Ginaton in Ang II-induced cardiac fibrosis showed that Ginaton treatment significantly reduced collagen deposition in the Ang II-infused heart compared to the PBS group (Figure 3A). Similarly, the number of α-SMA-positive myofibroblasts and the expression levels of α-SMA, collagen I, and collagen III mRNA were downregulated in Ginaton-treated mice compared to the PBS group (Figures 3B, C). As shown in Figure 3D, Ang II infusion induced increased TGF-β and Smad2 protein expression, while Ginaton treatment alleviated this response. These results suggested that Ginaton participates in Ang II-induced myocardial fibrosis through the TGF-β-Smad2 pathway.

### Ang II infusion induces M1 but not M2 phenotype macrophage activation, while Ginaton treatment downregulates the Ang II-induced inflammatory response

**FIGURE 4:**
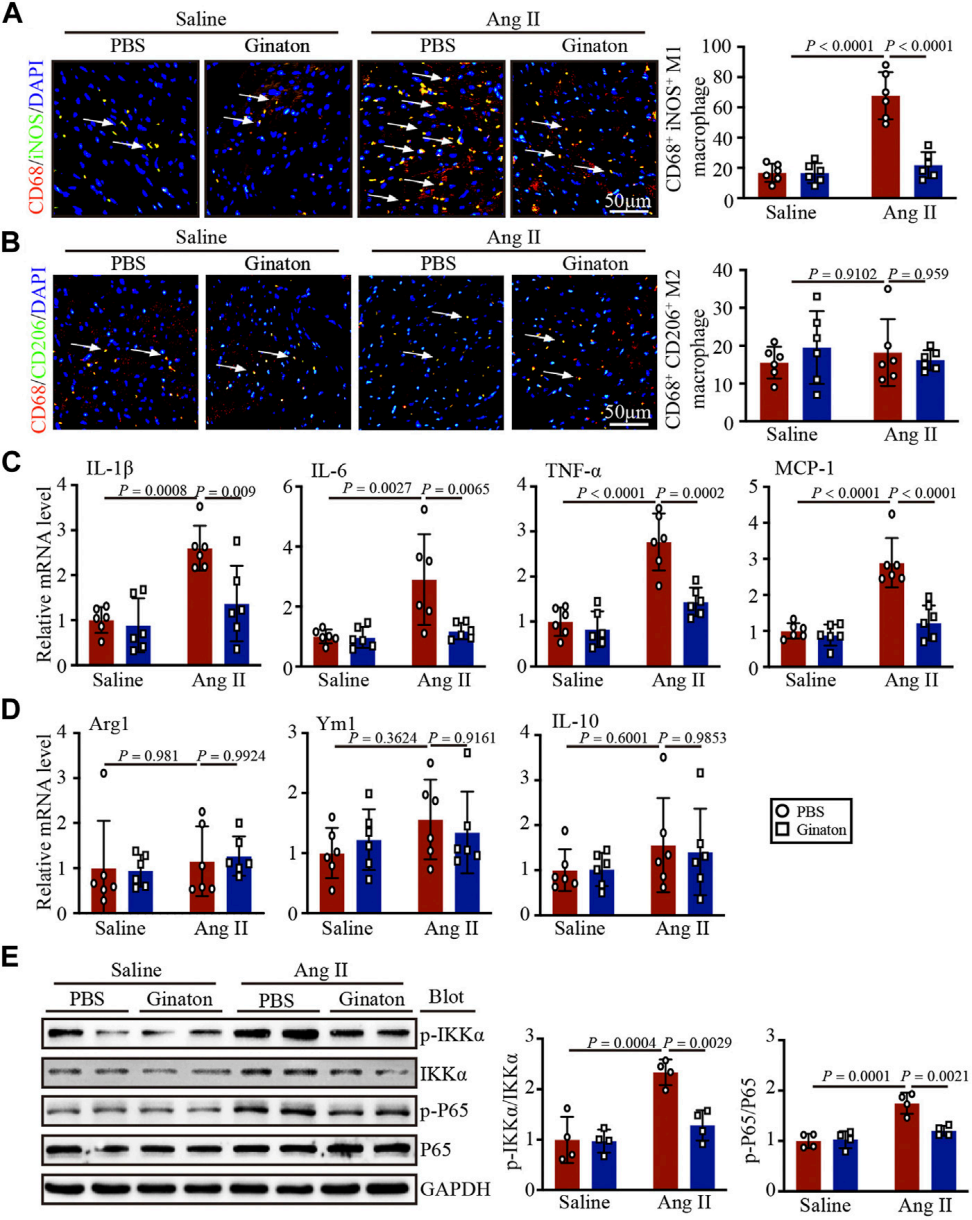
Ginaton decreases Ang II-induced M1 phenotype macrophage activation and inflammation through the NF-κB signaling pathway. Ginaton (300 mg/kg/day) or PBS (internal control) was administered intragastrically to 8-week-old C57BL/6J mice. Mice were then infused with Ang II (1,000 ng/kg/min) or saline for 14 days. **(A)** Immunofluorescence staining in cardiac sections with CD68 and iNOS antibodies (left, n = 6, scale bar = 50 μm) and quantification of CD68^+^ and iNOS^+^ macrophages in heart sections (right, n = 6). **(B)** Immunofluorescence staining of cardiac sections with CD68 and CD206 antibodies (left, n = 6, scale bar = 50 μm) and quantification of CD68^+^ and CD206^+^ macrophages in heart sections (right, n = 6). **(C)** qPCR analyses of IL-1β, IL-6, TNF-α, and MCP-1 mRNA levels (n = 6). **(D)** qPCR analyses of Arg1, Ym1, and IL-10 mRNA levels (n = 6). **(E)** Western blot analyses of p-IKKα, IKKα, p-P65, P65, and GAPDH expression in heart sections (left, n = 4) and the relative protein levels of each (right, n = 4).

To investigate the expression of different macrophage phenotypes in Ang II-infused hearts and the mechanism by which Ginaton improved Ang II-induced cardiac remodeling, we co-stained with the CD68 macrophage biomarker and the iNOS M1 phenotype macrophage biomarker. Immunofluorescent staining revealed that Ang II infusion induced high expression of iNOS^+^ M1 phenotype macrophages and that Ginaton reduced this Ang II-induced increase (Figure 4A). Furthermore, co-staining for the CD68 macrophage biomarker and the CD206 M2 phenotype macrophage biomarker showed no significant difference between Ang II infusion and Ginaton treatment on CD206^+^ M2 phenotype macrophages (Figure 4B). Similarly, Ang II infusion increased the mRNA levels of molecules associated with M1 phenotypic expression, including IL-1β, IL-6, TNF-α, and MCP-1, while Ginaton rescued this Ang II-induced inflammatory reaction. The levels of M2 phenotypic expression molecules, including Arg1, Ym1, and IL-10 mRNA levels, did not differ between the Ang II infusion and Ginaton treatment groups (Figures 4C, D). Moreover, IKKα and p65 phosphorylation were reduced in the Ginaton-treated group compared to the PBS-treated group (Figure 4E). These results suggested that Ginaton relieved Ang II-induced cardiac inflammation *via* the accumulation of M1 phenotype macrophages in the heart.

### Ginaton treatment reduces Ang II-induced M1 macrophage activation *in vitro*

**FIGURE 5:**
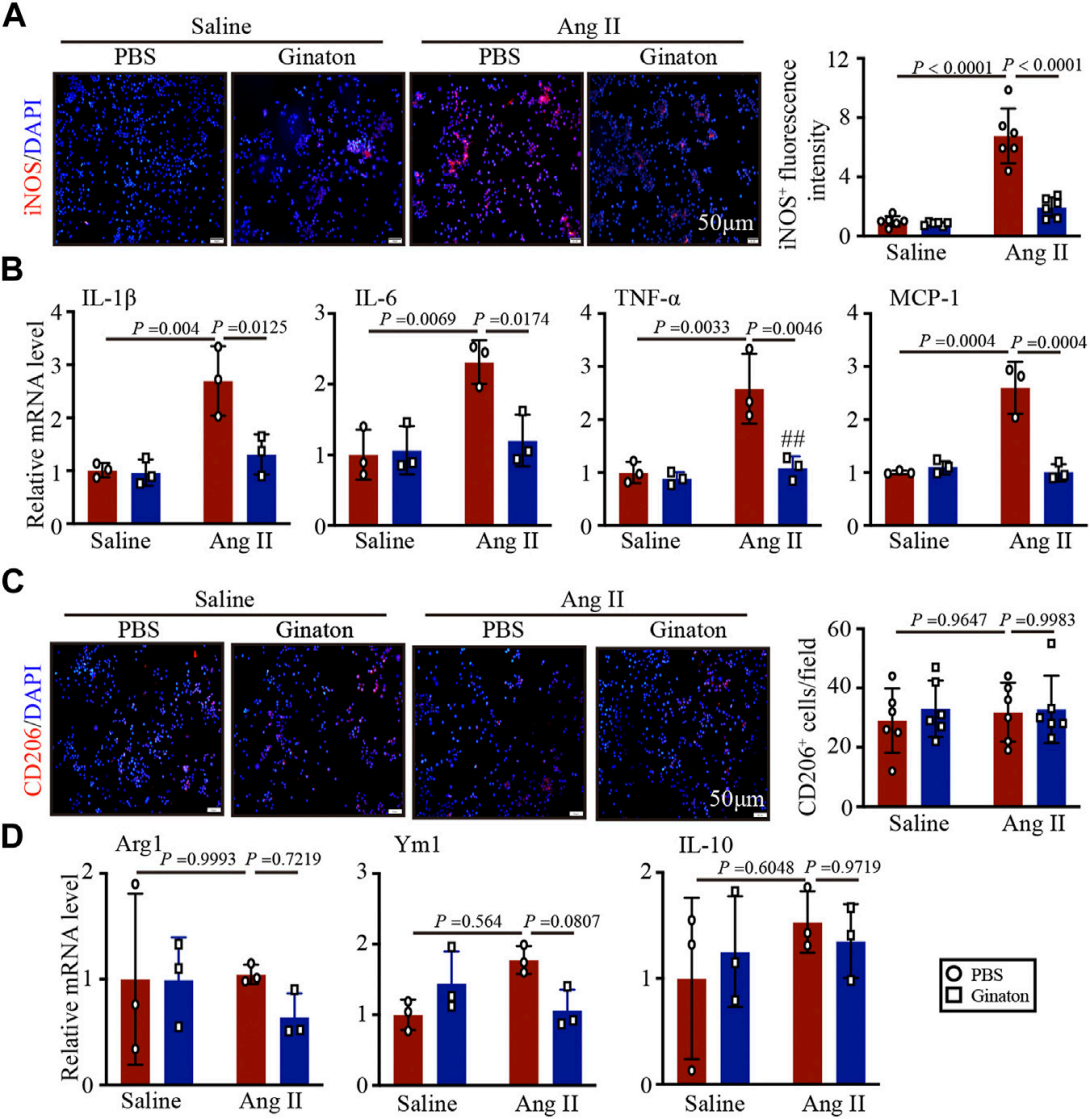
Application of Ginaton rescues the expression of M1 phenotype macrophages. Macrophages were treated with Ginaton (100 μg/ml) or PBS for 4 h and then with Ang II (100 ng/ml) or saline for 24 h. **(A)** Immunofluorescence staining in macrophages with CD68 and iNOS antibodies (left, n = 3, scale bar = 50 μm) and quantification of iNOS^+^ intensity (left, n = 3). **(B)** qPCR analyses of IL-1β, IL-6, TNF-α, and MCP-1 mRNA levels (n = 6). **(C)** Immunofluorescence staining of macrophages with CD68 and CD206 antibodies (left, n = 3, scale bar = 50 μm) and quantification of CD206^+^ intensity (left, n = 3). **(D)** qPCR analyses of Arg1, Ym1, and IL-10 mRNA levels (n = 6). The results are expressed as means ± SD; n, number of animals per group.

To confirm the effect of Ginaton on the activation of M1 phenotype macrophages induced by Ang II, we pretreated macrophages with Ginaton (100 μg/ml) or PBS for 4 h and with Ang II (100 nM) or saline for 24 h. We then co-stained the cells with the macrophage biomarker CD68 and the M1 phenotype macrophage biomarker iNOS. Immunofluorescence staining showed that Ang II treatment induced high expression of iNOS^+^ M1 phenotype macrophages and that Ginaton reduced the increase in iNOS^+^ M1 phenotype macrophages induced by Ang II *in vitro* (Figure 5A). Moreover, Ginaton reduced Ang II-induced mRNA expression of the M1 phenotype molecules IL-1β, IL-6, TNF-α, and MCP-1 (Figure 5B). Neither immunofluorescence staining nor qPCR results showed differences in M2 phenotype macrophages between the Ginaton and Ang II treatment groups (Figures 5C, D).

### Ginaton treatment inhibits macrophage adhesion and migration *in vitro*

**FIGURE 6:**
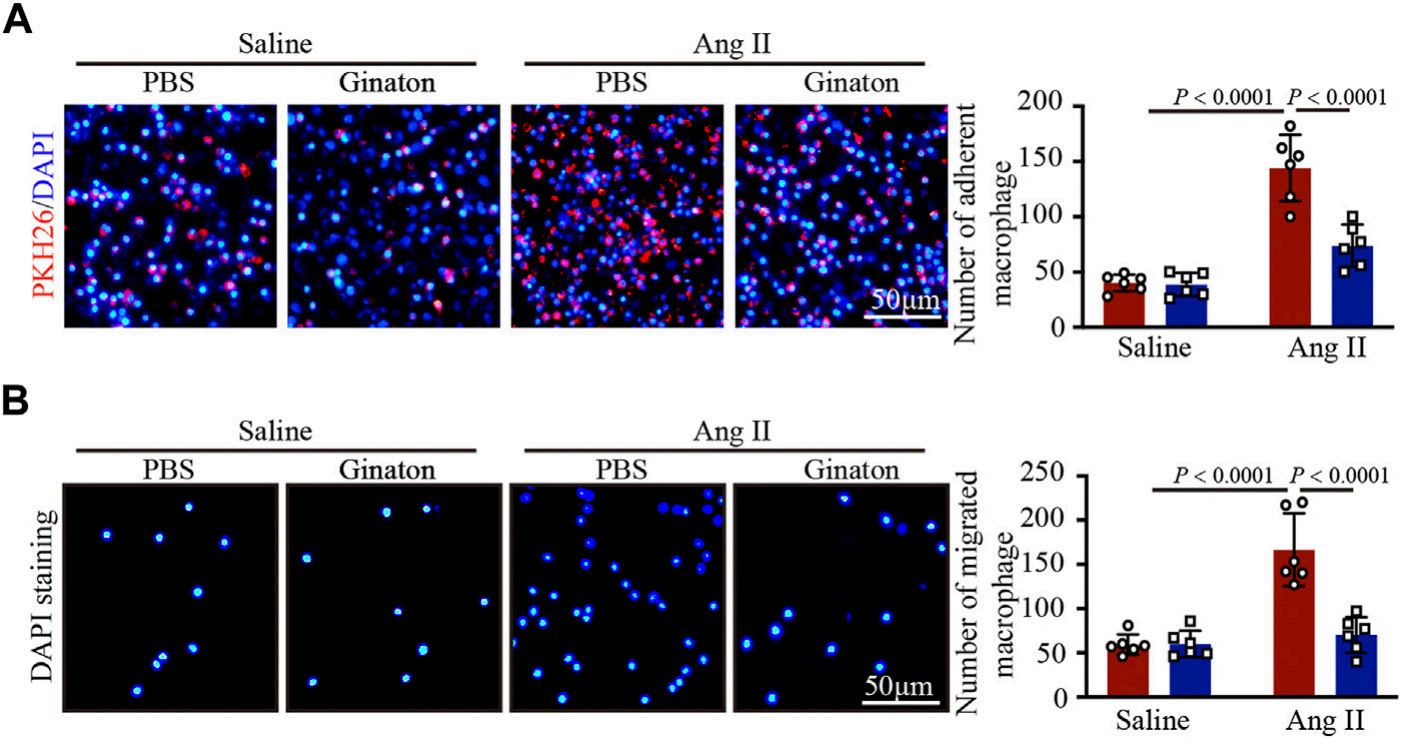
Ginaton reduces the increase of Ang II-induce macrophage adhesion and mitigation. **(A)** Human umbilical vein endothelial cells (HUVECs) were pretreated with Ginaton (100 μg/ml) or PBS for 4 h, and then with saline or Ang II (100 nM) for 24 h. PKH-26 staining of macrophages (left, n = 6, scale bar = 100 μm) and analysis of adherent cells (right, n = 3). **(B)** Macrophages (5 × 104) were added to the upper chamber on Transwell plate, and HUVEC-conditioned medium was added to the lower chamber of the plate. DAPI staining of macrophages (left, n = 6, scale bar = 100 μm) and analysis of mitigrated cells (right, n = 3). The results are expressed as mean ± SD; n, number of animals per group.

Coculture experiments of macrophages with HUVECs and Transwell assays showed that Ang II treatment increased macrophage adhesion and migration to HUVECs, an effect that was inhibited by Ginaton (Figures 6A, B). The results showed that Ginaton could reduce the adhesion and migration of macrophages to HUVECs.

## Discussion

**FIGURE 7:**
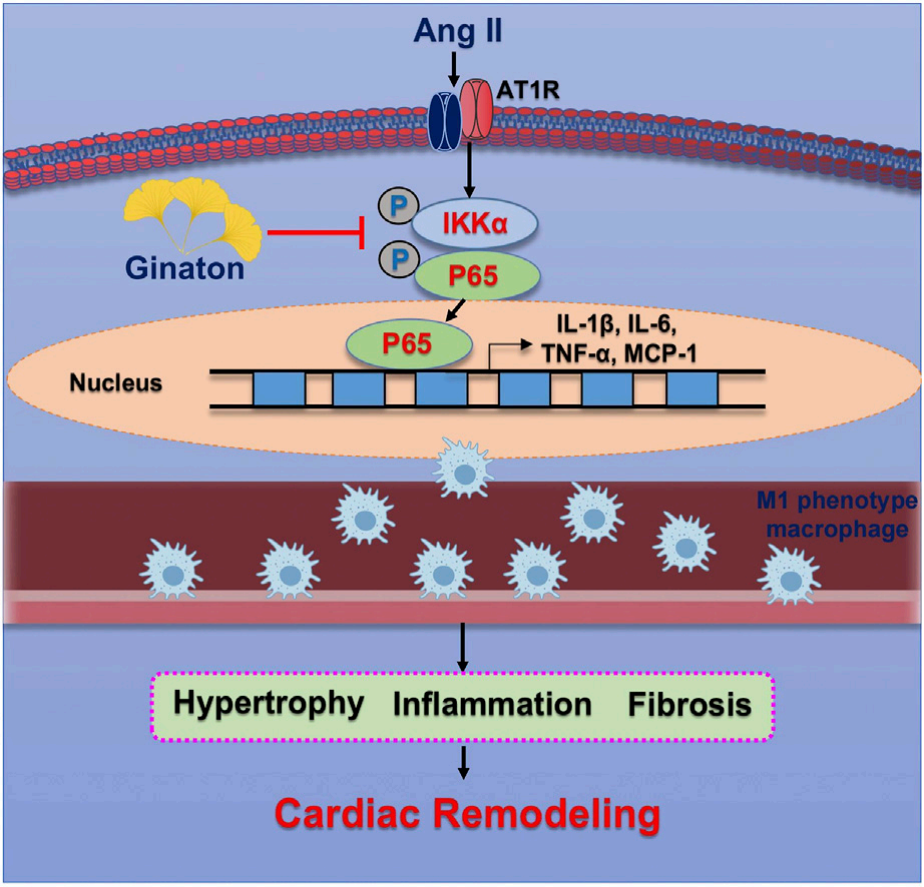
Operational representation of the method through which Ginaton controls cardiac remodeling caused by Ang II. By encouraging M1 phenotype macrophages to adhere to the endothelium and infiltrate the heart, Ang II infusion increases NF-κB activation, which in turn causes cardiomyocyte hypertrophy, fibrosis, and subsequent cardiac remodeling. Ginaton administration successfully reverses these effects.

The results of the present study demonstrated the protective effect of Ginaton on Ang II-induced cardiac remodeling in mice. After 14 days of Ang II infusion, the mice showed significantly elevated SBP, upregulated expression of M1 phenotype macrophages, monocyte/macrophage adherence to vascular endothelial cells, and subsequent migration to the heart tissue across the endothelium, producing proinflammatory cytokines, whose activation led to multiple signaling pathways of hypertensive cardiac remodeling. In contrast, Ginaton alleviated Ang II-induced hypertension, the inflammatory response, and monocyte/macrophage adhesion and migration, which might provide a new perspective for the treatment of hypertension (Figure 7).

Hypertension is a major risk factor for heart failure and is characterized by chronic low-grade inflammation, whichleads to poor cardiac remodeling (Mikolajczyk et al., 2021). Although macrophages play a critical role in cardiac remodeling, dysregulated macrophage polarization between the proinflammatory M1 and anti-inflammatory M2 phenotypes promotes excessive inflammation and cardiac damage (Gullestad et al., 2012; Varol et al., 2015). M1 phenotype macrophages express many proinflammatory mediators, including TNF-α, IL-1, IL-6, and iNOS, while M2 phenotype macrophages express molecules including Arg1, IL-10, Ym1, and CD206 (Lavine et al., 2014; Kologrivova et al., 2021). Many key transcription factors are involved in macrophage polarization, such as signal transducers and activators of transcription (STATs), interferon regulatory factors (IRFs), nuclear factors (NF-κB), activator protein (AP) 1, and peroxisome proliferator-activated receptor (PPAR)-γ (Ohmori et al., 1997; Bouhlel et al., 2007; Odegaard et al., 2007; Satoh et al., 2010; Krausgruber et al., 2011; Lawrence et al., 2011; Oeckinghaus et al., 2011; Schonthaler et al., 2011). NF-κB is a family of transcription factors involved in many biological processes, including the immune response, inflammation, cell growth, survival, and development. NF-κB protein is usually expressed by a series of inhibitors, including κBα immobilized in the cytoplasm. The typical NF-κB activation pathway depends on IκBs, particularly IκBα-inducible degradation (Mitchell et al., 2016). The degradation of IκBα is mediated through its phosphorylation by IκB kinase α (IKKα) (Behar et al., 2013). The present study focused on macrophage phenotypes and their impacts on the outcome of hypertensive cardiac remodeling. We found that following infusion with Ang II, macrophages tended to switch to an M1 phenotype and expressed higher levels of iNOS and more pro-inflammatory cytokines such as IL-1β, IL-6, TNF-α, and MCP-1. Ang II infusion also increased p-IKKα and P-p65 expression. In further investigations, we examined the expression of CD206^+^ M2 phenotype macrophages and proinflammatory cytokines such as Arg1, Ym1, and IL-10. We found that Ang II infusion did not significantly increase the expression of M2 phenotype macrophages. These results suggested that Ang II infusion activated the proinflammatory response in the heart and that the activation of macrophage adhesion and migration was a major cause of cardiac remodeling.

Ginaton, a natural product extracted from *Ginkgo biloba* leaves, has been widely used worldwide and has therapeutic effects on a variety of diseases (Tian et al., 2017). Recent studies have found that treatment with Ginaton alleviated renal interstitial fibrosis through the TGF-β pathway and that Ginaton also reduced TNF-α, IL-1β, and 5-hydroxytryptamine levels in the hippocampus of mice with heart failure (Zhang et al., 2019). In the present study, after treatment with Ginaton, Ang II-induced hypertrophy and fibrosis were both alleviated. Moreover, we detected the expression of macrophages with different phenotypes in Ang II-infused hearts. The results showed markedly decreased levels of M1 phenotype macrophages and proinflammatory cytokines after Ginaton treatment, with no obvious change in M2 phenotype macrophages or proinflammatory cytokines. Ginaton also reduced the Ang II-induced activation of macrophage adhesion and migration. These results indicate that Ginaton may offer an approach for the treatment of hypertensive cardiac remodeling.

In conclusion, our results showed that Ginaton treatment inhibited Ang II-induced M1 phenotype macrophage activation, adhesion and mitigation, and the inflammatory response, leading to impaired and dysfunctional hypertension and cardiac remodeling. Therefore, Ginaton may be a powerful treatment for heart disease.

## Data Availability

The original contributions presented in the study are included in the article/Supplementary Material; further inquiries can be directed to the corresponding authors.
