# Supplementary material for: Ginaton reduces M1-polarized macrophages in hypertensive cardiac remodeling via NF-κB signaling
Source: Front Pharmacol. 2023 Mar 13;14:1104871. doi: 10.3389/fphar.2023.1104871 (PMC10040779; doi:10.3389/fphar.2023.1104871)
Supplement: Supplementary file 1 [file Table1.DOCX]

**Table S1.** Echocardiographic parameters of wild-type mice treatment with Ginaton or PBS control after Ang II infusion for 14days.

| Parameter | Saline+PBS | Saline+Ginaton | Ang II+PBS | Ang II+Ginaton |
| --- | --- | --- | --- | --- |
| HR (mm/Hg) | 603±30.22 | 621±17.99 | 625±36.62 | 617±33.72 |
| EF % | 61.68±6.67 | 63.47±12.35 | 77.81±3.02^***^ | 66.62±8.01^#^ |
| FS % | 32.58±5.17 | 34.02±7.12 | 45.10±2.95^***^ | 35.93±5.85^##^ |
| LVAW; d (mm) | 0.81±0.11 | 0.84±0.17 | 1.00±0.08^**^ | 0.86±0.06^#^ |
| LVAW; s (mm) | 1.27±0.14 | 1.26±0.23 | 1.64±0.04^***^ | 1.46±0.08^##^ |
| LVID; d (mm) | 3.67±0.14 | 3.72±0.69 | 2.76±0.14^***^ | 3.38±0.29^###^ |
| LVID; s (mm) | 2.52±0.28 | 2.46±0.49 | 1.54±0.18^***^ | 2.05±0.33^##^ |
| LVPW; d (mm) | 0.76±0.07 | 0.80±0.17 | 1.11±0.16^***^ | 0.86±0.12^##^ |
| LVPW; s (mm) | 1.06±0.12 | 1.10±0.24 | 1.54±0.11^***^ | 1.29±0.36^#^ |

Values: means ± SD (n = 8);

**p<0.01 and ***p<0.001 vs. Saline+PBS; ^#^p<0.05, ^##^p<0.01 and ^###^p<0.001 vs. Ang II+PBS.

HR, heart rate; EF, ejection fraction; FS, fractional shortening; LVAW; d, left ventricular anterior wall at end-diastole; LVAW; s, left ventricular anterior wall at end-systole; LVID; d, left ventricular internal dimension at end-diastole; LVID; s, left ventricular internal dimension at end-systole; LVPW; d, left ventricular posterior wall at end-diastole; LVPW; s, left ventricular posterior wall at end-systole.
