# Supplementary material for: Ginaton reduces M1-polarized macrophages in hypertensive cardiac remodeling via NF-κB signaling
Source: Front Pharmacol. 2023 Mar 13;14:1104871. doi: 10.3389/fphar.2023.1104871 (PMC10040779; doi:10.3389/fphar.2023.1104871)
Supplement: Supplementary file 2 [file Table2.DOCX]

**Table S2.** Primers used for quantitative real-time PCR analysis

| Gene | Forward primer (5’-3’) | Reverse primer (5’-3’) |
| --- | --- | --- |
| ANF | CACAGATCTGATGGATTTCAAGA | CCTCATCTTCTACCGGCATC |
| BNP | GAAGGTGCTGTCCCAGATGA | CCAGCAGCTGCATCTTGAAT |
| Collagen I | GAGTACTGGATCGACCCTAACCA | GACGGCTGAGTAGGGAACACA |
| Collagen III | TCCCCTGGAATCTGTGAATC | TGAGTCGAATTGGGGAGAAT |
| α-SMA | TCCTGACGCTGAAGTATCCGATA | GGCCACACGAAGCTCGTTAT |
| IL-1β | TGCCACCTTTTGACAGTGATG | TGATGTGCTGCTGCGAGATT |
| IL-6 | TGATGGATGCTACCAAACTGGA | TGTGACTCCAGCTTATCTCTTGG |
| TNF-α | CAGGCGGTGCCTATGTCTC | CGATCACCCCGAAGTTCAGTAG |
| MCP-1 | TAAAAACCTGGATCGGAACCAAA | GCATTAGCTTCAGATTTACGGGT |
| Arg1 | CTCCAAGCCAAAGTCCTTAGAG | GGAGCTGTCATTAGGGACATCA |
| Ym1 | CAGGTCTGGCAATTCTTCTGAA | GTCTTGCTCATGTGTGTAAGTGA |
| IL-10 | CTTACTGACTGGCATGAGGATCA | GCAGCTCTAGGAGCATGTGG |
| GAPDH | GGTTGTCTCCTGCGACTTCA | GGTGGTCCAGGGTTTCTTACTC |

ANF, atrial natriuretic factor; BNP, brain natriuretic factor; α-SMA, α-smooth muscle actin; IL-1β, interleukin 1 beta; IL-6, interleukin 6; TNF-α, tumor necrosis factor alpha; MCP-1, monocyte chemotactic protein 1; Arg1, arginase 1; Ym1, chitinase-like protein 3; IL-10, interleukin 10; GAPDH, glyceraldehyde 3-phosphate dehydrogenase.
