# Supplementary material for: Ginaton reduces M1-polarized macrophages in hypertensive cardiac remodeling via NF-κB signaling
Source: Front Pharmacol. 2023 Mar 13;14:1104871. doi: 10.3389/fphar.2023.1104871 (PMC10040779; doi:10.3389/fphar.2023.1104871)
Supplement: Supplementary file 3 [file DataSheet1.PDF]

Figure 1

A SBP

	WS+PBS						WS+Ginaton					
-1	103	98	97	83	100	99	95	101	102	107	97	96
1	102	94	112	85	89	105	101	104	102	101	99	94
3	92	98	100	101	96	88	98	99	96	101	105	99
5	91	90	87	96	100	110	94	89	107	92	97	80
7	96	108	103	92	98	97	113	93	105	92	94	94
9	98	94	93	99	101	98	104	109	103	94	100	94
11	92	104	104	103	99	100	103	110	96	93	103	100
13	100	105	94	97	104	103	102	109	105	107	94	100

WA+PBS						WA+Ginaton					
106	93	105	109	109	103	98	108	112	108	107	87
108	114	129	145	107	104	120	105	99	103	111	105
125	141	137	133	122	104	115	113	102	91	108	116
141	135	133	131	127	125	104	108	99	109	112	117
135	144	134	148	130	127	119	117	95	106	104	101
161	152	145	138	128	133	111	108	102	111	103	99
153	156	168	147	132	127	121	113	124	100	100	103
160	140	151	174	143	132	113	109	113	122	97	97

B Heart rate

WS+PBS	WS+Ginaton	WA+PBS	WA+Ginaton
642	607	533	617
591	597	655	682
620	608	665	578
659	622	628	619
580	621	626	611
592	641	611	609
564	614	626	565
572	660	655	658

C M-mode echocardiographic

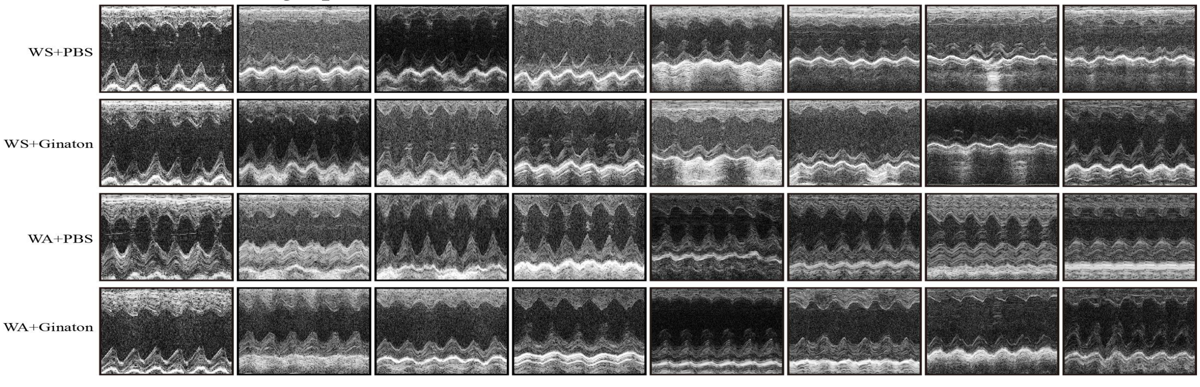

D

EF%	FS%	LVAW;d	LVAW;	LVID;d	LVID;s	LVPW;d	LVPW;s

s

WS+PBS 1	59.81	28.97	0.74	1.50	3.79	3.07	0.66	0.88
WS+PBS 2	58.38	30.35	0.82	1.26	3.91	2.72	0.74	0.99
WS+PBS 3	61.97	32.61	0.84	1.21	3.58	2.41	0.72	0.99
WS+PBS 4	67.36	36.42	0.76	1.13	3.36	2.14	0.70	1.03
WS+PBS 5	55.49	28.18	0.68	1.05	3.52	2.53	0.82	1.13
WS+PBS 6	52.51	26.16	0.89	1.30	3.34	2.47	0.76	1.01
WS+PBS 7	76.07	44.10	1.05	1.48	3.79	2.12	0.89	1.32
WS+PBS 8	63.22	33.82	0.74	1.23	4.03	2.66	0.76	1.11
**Mean**	**61.85**	**32.58**	**0.81**	**1.27**	**3.67**	**2.52**	**0.76**	**1.06**
**SD**	**6.67**	**5.17**	**0.11**	**0.14**	**0.23**	**0.28**	**0.07**	**0.12**
WS+Ginaton1	56.65	28.96	0.89	1.26	3.56	2.53	0.89	1.05
WS+Ginaton2	69.29	38.22	0.72	1.26	3.71	2.29	0.66	1.09
WS+Ginaton3	70.48	39.15	1.03	1.44	3.68	2.24	0.89	1.28
WS+Ginaton4	63.29	33.60	0.72	1.26	3.65	2.42	0.85	1.37
WS+Ginaton5	63.78	34.16	0.82	1.28	3.93	2.59	0.66	0.91
WS+Ginaton6	57.00	29.26	0.91	1.15	3.66	2.59	0.72	0.86
WS+Ginaton7	54.46	27.91	0.74	1.19	4.18	3.01	0.78	0.99
WS+Ginaton8	72.83	40.91	0.91	1.23	3.42	2.02	0.97	1.28
**Mean**	**63.47**	**34.02**	**0.84**	**1.26**	**3.72**	**2.46**	**0.80**	**1.10**
**SD**	**12.35**	**7.12**	**0.17**	**0.23**	**0.69**	**0.49**	**0.17**	**0.24**
WA+PBS 1	83.89	51.53	0.99	1.63	2.57	1.54	1.17	1.75
WA+PBS 2	77.15	44.22	0.97	1.67	2.86	1.59	1.03	1.36
WA+PBS 3	77.19	44.30	0.91	1.69	2.90	1.61	1.03	1.42
WA+PBS 4	76.10	44.16	1.11	1.59	2.74	1.23	1.17	1.58
WA+PBS 5	79.55	46.15	1.07	1.63	2.53	1.36	1.46	1.58
WA+PBS 6	74.73	42.33	0.91	1.58	2.80	1.83	0.88	1.52
WA+PBS 7	73.89	41.33	1.13	1.61	2.92	1.71	0.99	1.52
WA+PBS 8	79.94	46.81	0.93	1.69	2.74	1.46	1.17	1.58
**Mean**	**77.81**	**45.10**	**1.00**	**1.64**	**2.76**	**1.54**	**1.11**	**1.54**
**SD**	**3.02**	**2.95**	**0.08**	**0.04**	**0.14**	**0.18**	**0.16**	**0.11**
WA+Ginaton1	57.31	28.95	0.89	1.34	3.15	2.22	0.88	1.30
WA+Ginaton2	76.72	43.75	0.99	1.48	3.17	1.69	0.72	1.26
WA+Ginaton3	73.77	40.65	0.80	1.50	3.17	2.14	0.93	1.30
WA+Ginaton4	68.11	37.16	0.82	1.46	3.73	2.28	0.70	1.28
WA+Ginaton5	54.29	27.37	0.82	1.34	3.44	2.51	0.88	1.34
WA+Ginaton6	75.17	42.11	0.88	1.58	3.83	1.46	1.11	1.52
WA+Ginaton7	59.92	30.61	0.82	1.48	2.98	1.87	0.78	1.15
WA+Ginaton8	67.68	36.81	0.88	1.52	3.56	2.28	0.88	1.40
**Mean**	**66.62**	**35.93**	**0.86**	**1.46**	**3.38**	**2.05**	**0.86**	**1.29**
**SD**	**8.01**	**5.85**	**0.06**	**0.08**	**0.29**	**0.33**	**0.12**	**0.36**

Figure 2

A H&E

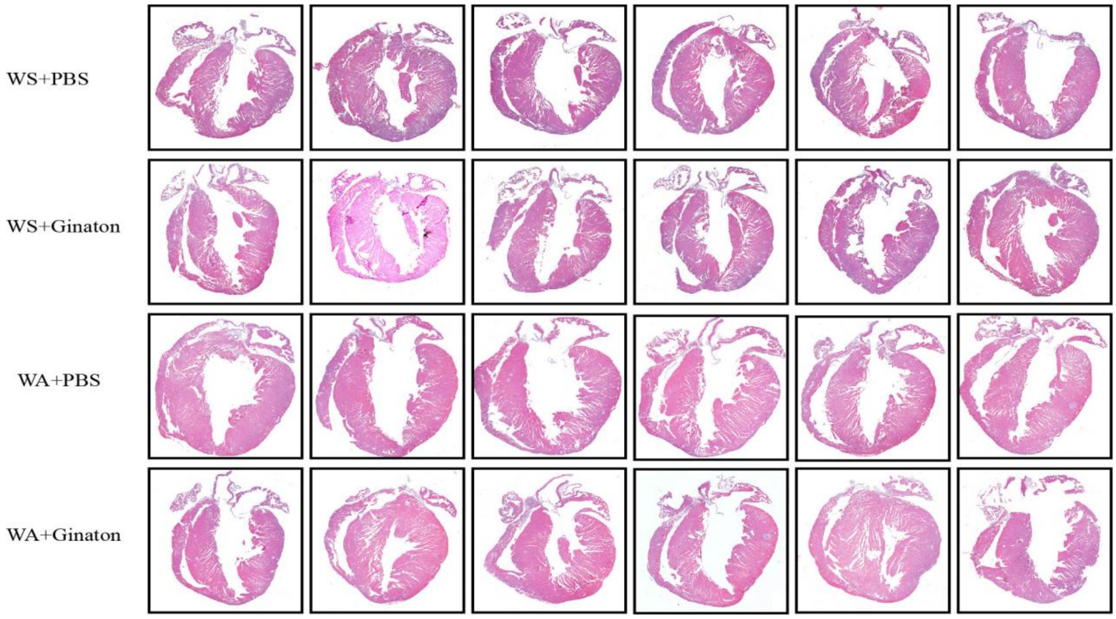

HW/BW

Group	1	2	3	4	5	6	7
WS+PBS	4.79	4.68	4.85	5.06	4.65	4.58	4.36
WS+Ginaton	4.49	4.82	4.92	4.92	4.76	4.68	4.34
WA+PBS	6.44	5.43	5.98	5.79	6.29	5.92	5.44
WA+Ginaton	4.78	4.75	4.88	4.99	5.21	4.59	5.28

HW/TL

Group	1	2	3	4	5	6	7
WS+PBS	7.08	7.35	7.53	7.49	6.8	6.93	6.42
WS+Ginaton	6.49	7.10	6.98	6.92	7.03	6.82	6.75
WA+PBS	7.57	8.04	8.40	8.25	8.62	8.5	7.98
WA+Ginaton	6.54	7.30	7.32	7.54	7.96	7.37	7.73

B WGA

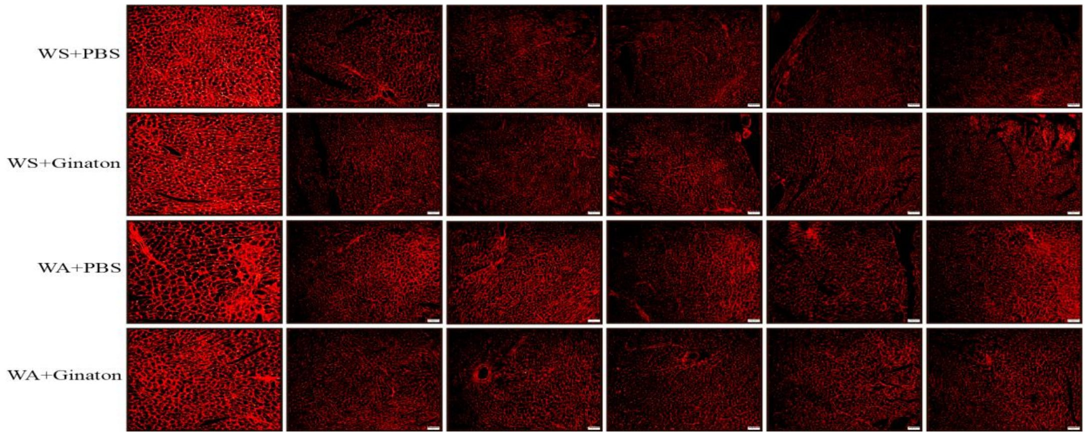

Group	1	2	3	4	5	6
WS+PBS	238	208	190	209	220	203

WS+Ginaton	200	243	204	207	194	206
WA+PBS	532	445	414	510	484	568
WA+Ginaton	256	201	206	232	216	240

C ANF BNP

Group	1	2	3	4	5	6
WS+PBS	1.06	1.02	1.16	0.88	0.90	1.09
WS+Ginaton	0.87	1.21	0.75	1.17	0.41	1.13
WA+PBS	6.32	4.21	2.01	2.87	2.92	6.06
WA+Ginaton	2.01	0.67	1.76	2.56	1.71	2.49
Group	1	2	3	4	5	6
WS+PBS	0.49	0.96	0.75	0.73	1.43	1.61
WS+Ginaton	0.75	0.74	1.42	1.83	0.7	1.12
WA+PBS	0.66	3.34	2.66	2.95	4.03	2.98
WA+Ginaton	2.25	1.41	1.08	1.26	2.29	1.24

D WB

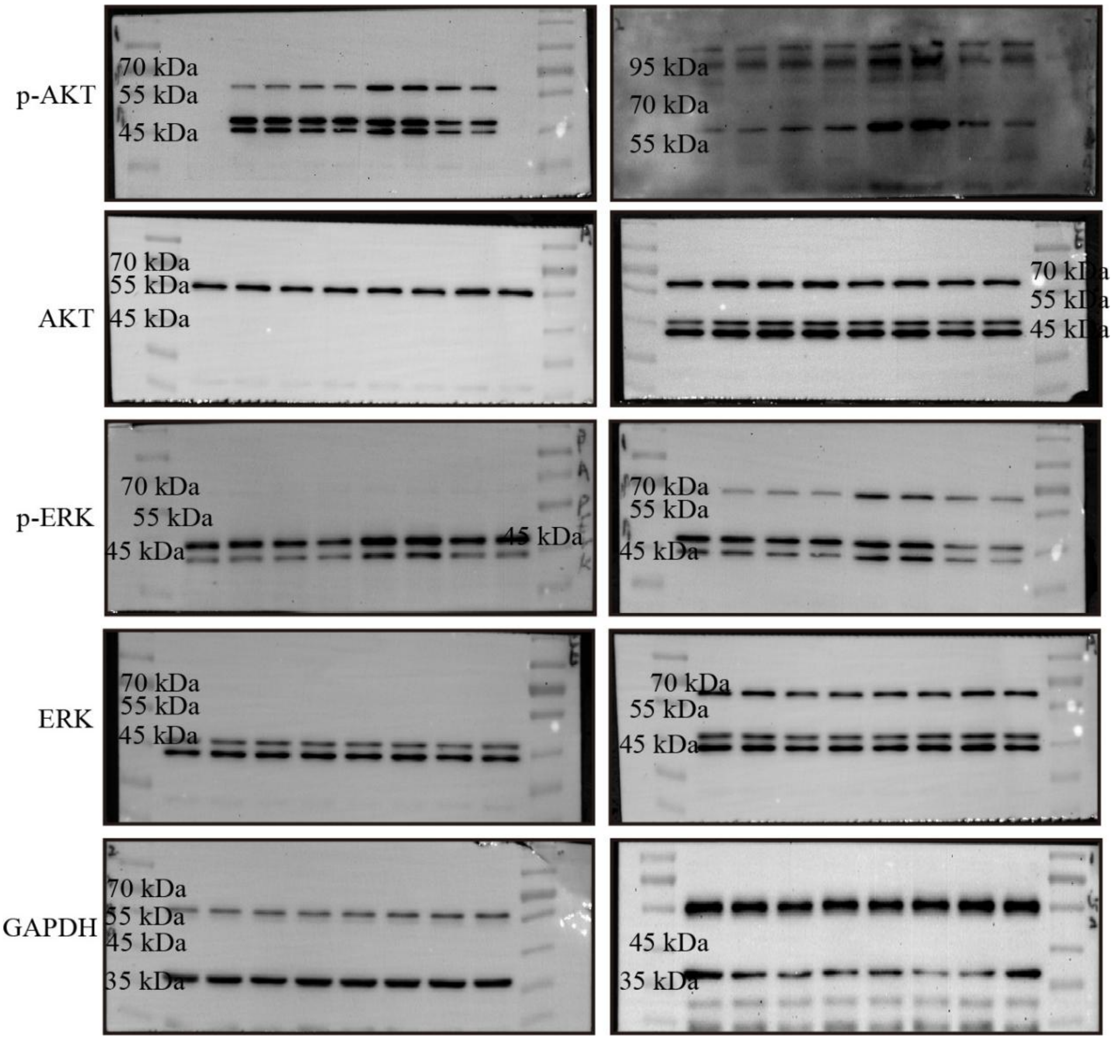

Group	1	2	3	4

p-AKT/AKT

WS+PBS	0.86	1.28	0.90	0.93
WS+Ginaton	1.21	1.33	1.14	1.21
WA+PBS	3.72	3.31	3.16	3.28
WA+Ginaton	1.50	1.91	0.86	1.17

P-ERK/ERK

Group	1	2	3	4
WS+PBS	0.81	0.96	1.14	1.08
WS+Ginaton	0.94	0.80	0.94	0.9
WA+PBS	1.55	1.57	1.93	1.84
WA+Ginaton	1.08	1.11	0.88	0.92

Figure 3

A Masson

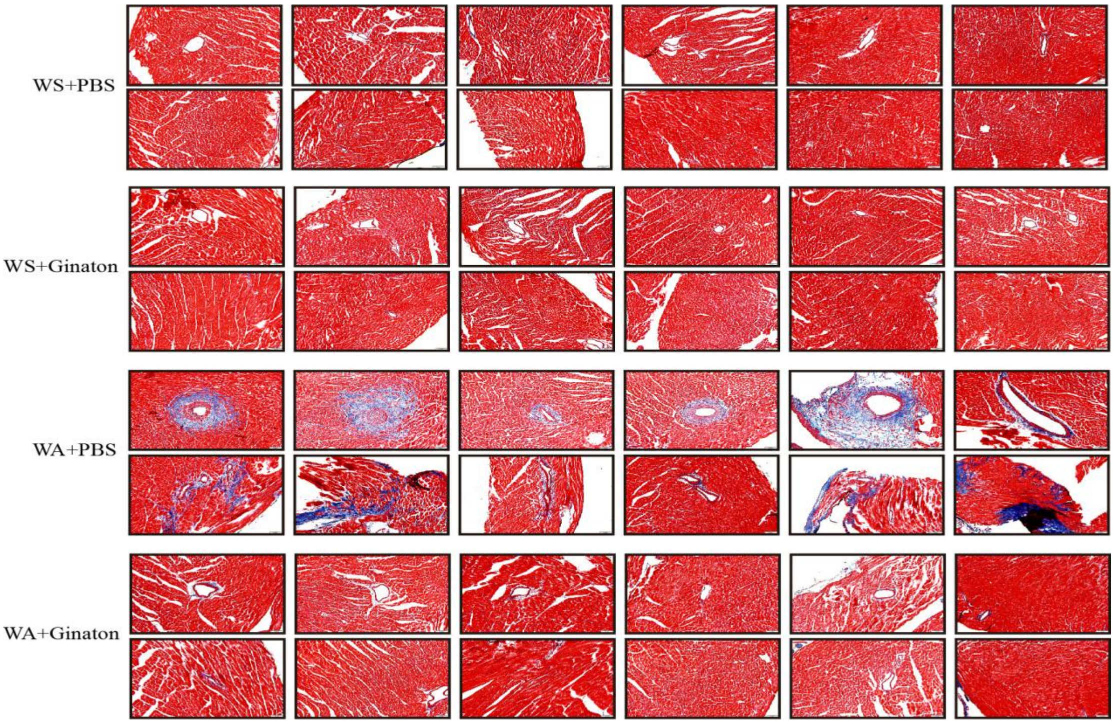

Group	1	2	3	4	5	6
WS+PBS	1.06	0.98	0.89	1.20	1.12	0.75
WS+Ginaton	0.76	1.01	0.82	1.35	1.5	0.64
WA+PBS	3.42	3.73	2.23	3.23	2.61	2.41
WA+Ginaton	1.27	1.32	0.95	1.43	1.24	1.03

B α-SMA

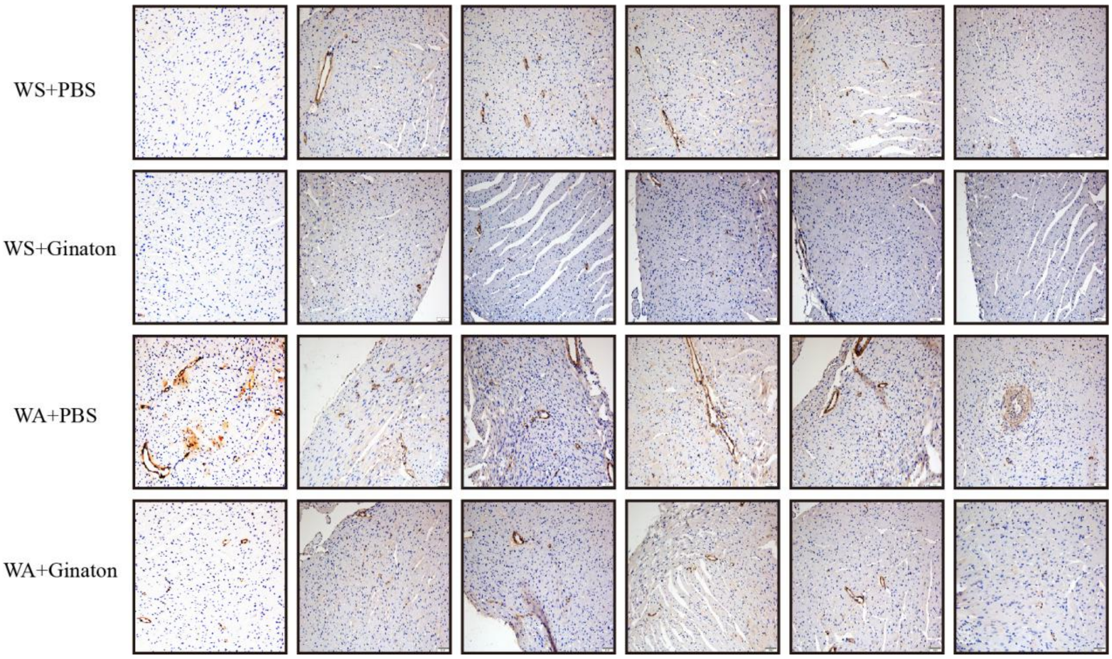

Group	1	2	3	4	5	6
WS+PBS	1.20	0.74	1.06	1.08	1.51	0.40
WS+Ginaton	1.01	0.80	1.19	0.92	1.17	1.01
WA+PBS	2.78	2.51	1.96	2.49	2.03	1.31
WA+Ginaton	1.11	1.08	1.13	1.33	1.38	0.75

C qPCR

α-SMA

Group	1	2	3	4	5	6
WS+PBS	0.49	0.84	1.29	1.63	1.45	0.30
WS+Ginaton	1.03	0.64	0.85	0.80	0.71	0.90
WA+PBS	3.73	2.71	2.99	2.04	6.63	2.79
WA+Ginaton	1.70	1.40	1.91	1.76	1.68	2.01

Collagen I

Group	1	2	3	4	5	6
WS+PBS	0.38	1.34	1.82	0.89	1.32	0.29
WS+Ginaton	2.13	0.36	0.95	0.71	0.46	0.91
WA+PBS	2.08	2.69	2.09	3.29	2.60	1.33
WA+Ginaton	1.28	0.96	1.58	1.15	0.62	1.34

Collagen III

Group	1	2	3	4	5	6
WS+PBS	0.15	1.08	1.89	0.93	1.48	0.48
WS+Ginaton	0.73	0.11	0.94	0.73	0.34	2.36
WA+PBS	2.94	3.23	5.08	3.14	2.23	2.80
WA+Ginaton	2.51	0.71	1.55	1.06	2.10	2.09

D WB

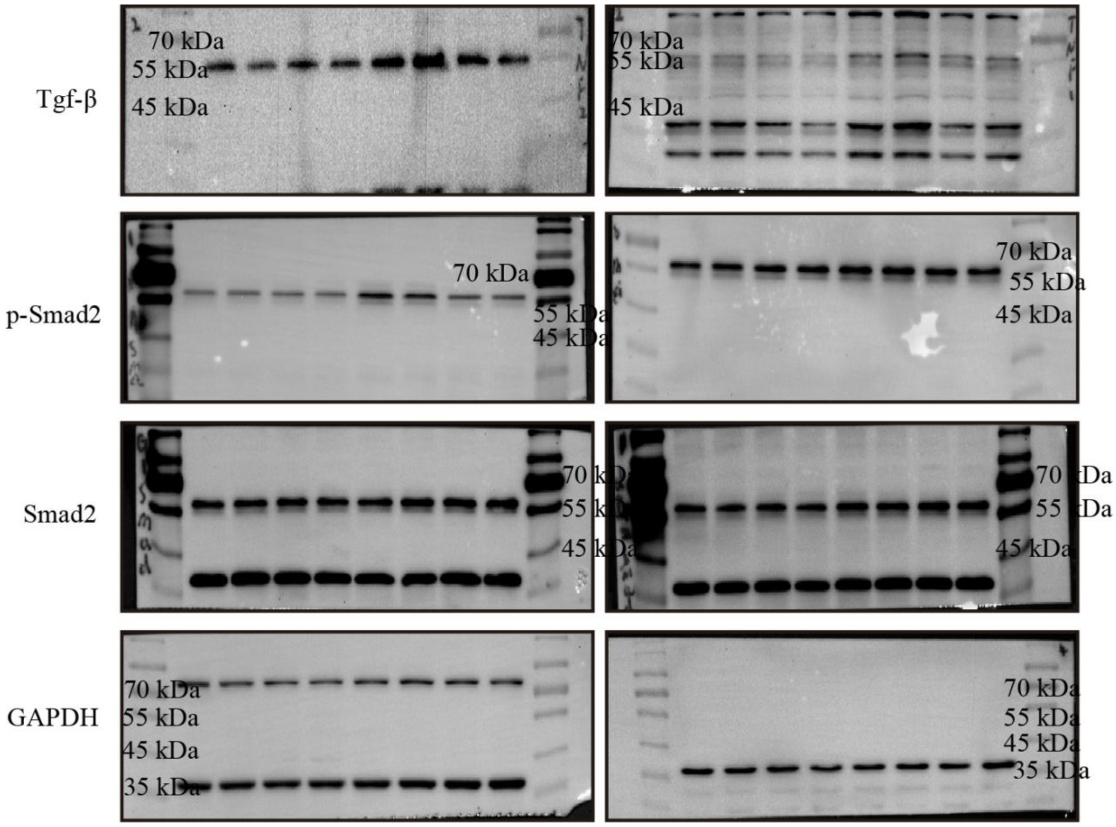

Tgf-β

Group	1	2	3	4
WS+PBS	1.17	0.62	0.89	1.30
WS+Ginaton	1.25	0.99	0.90	0.62
WA+PBS	2.33	3.20	1.94	3.21
WA+Ginaton	1.82	1.15	1.46	1.67

p-Smad2/Smad2

Group	1	2	3	4
WS+PBS	0.87	0.81	1.07	1.24
WS+Ginaton	0.79	0.80	1.08	1.09
WA+PBS	1.93	1.87	2.24	2.29
WA+Ginaton	0.88	0.93	1.42	1.25

Figure 4

A CD68/iNOS/DAPI

Group	1	2	3	4	5	6
WS+PBS	15	18	22	24	13	8
WS+Ginaton	12	8	14	21	26	18
WA+PBS	53	70	49	82	88	64
WA+Ginaton	18	28	23	35	12	15

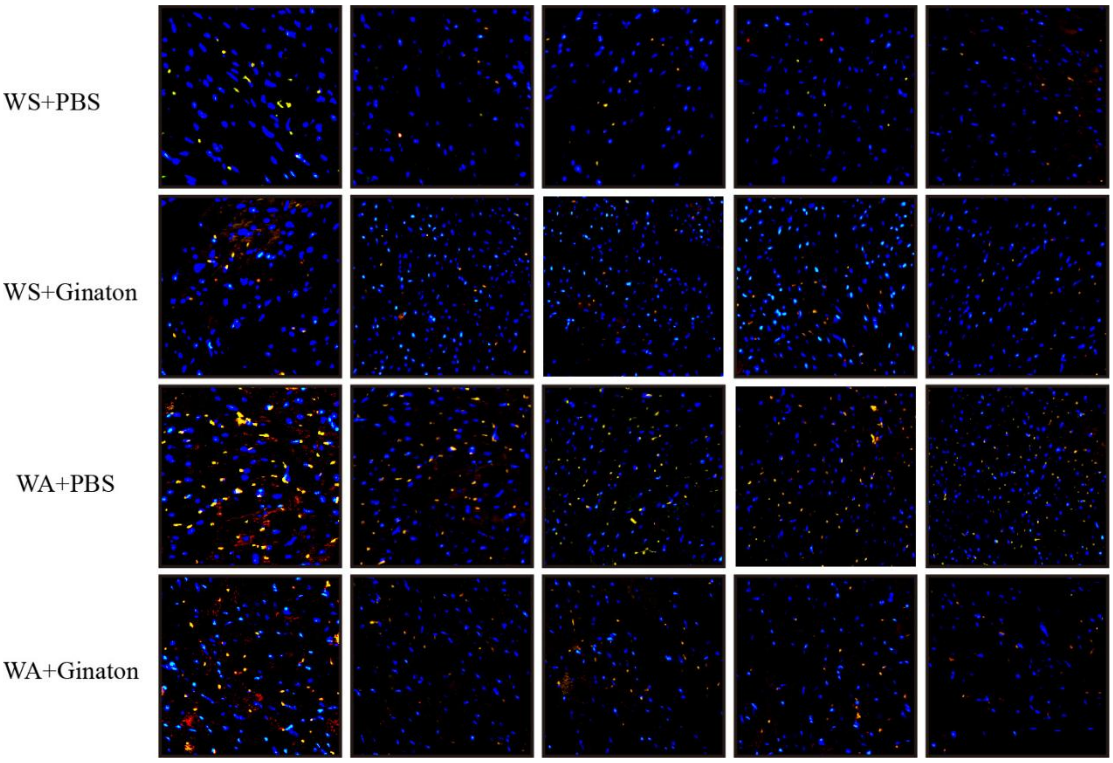

B CD68/CD206/DAPI

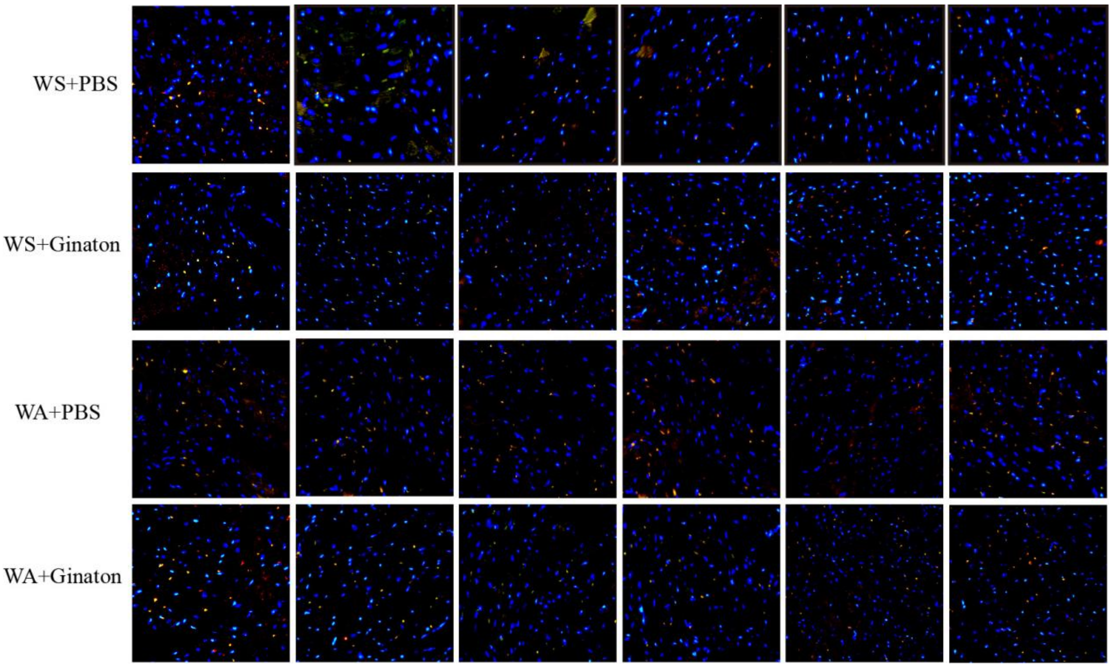

Group	1	2	3	4	5	6
WS+PBS	13	9	15	17	18	21
WS+Ginaton	11	22	7	18	26	33
WA+PBS	16	20	12	15	35	11
WA+Ginaton	14	18	15	17	20	13

C qPCR

IL-1β

Group	1	2	3	4	5	6
WS+PBS	1.06	1.25	1.31	1.04	0.86	0.52
WS+Ginaton	1.68	0.32	0.41	0.6	0.7	1.60
WA+PBS	2.44	3.53	2.64	2.19	2.63	2.17
WA+Ginaton	1.16	0.36	1.23	0.85	1.87	2.76

IL-6

Group	1	2	3	4	5	6
WS+PBS	0.627	1.159	1.311	0.939	1.009	0.973
WS+Ginaton	1.314	1.461	0.897	0.868	0.583	0.688
WA+PBS	1.853	5.238	2.041	3.65	1.108	3.517
WA+Ginaton	1.519	0.885	1.488	1.194	1.04	0.95

TNF-α

Group	1	2	3	4	5	6
WS+PBS	0.765	1.296	0.918	1.152	1.337	0.531
WS+Ginaton	1.441	0.672	0.736	0.53	0.415	1.182
WA+PBS	3.344	1.795	2.354	3.514	2.848	2.765
WA+Ginaton	1.659	1.452	1.073	1.179	1.923	1.334

MCP-1

Group	1	2	3	4	5	6
WS+PBS	0.75	0.881	0.863	1.105	1.07	1.329
WS+Ginaton	1.161	0.852	0.844	1.192	0.374	0.881
WA+PBS	2.448	2.461	2.821	2.823	2.551	4.245
WA+Ginaton	1.138	0.628	1.324	1.867	0.744	1.628

D qPCR

Arg1

Group	1	2	3	4	5	6
WS+PBS	0.28	0.52	3.11	0.59	0.84	0.66
WS+Ginaton	0.75	0.6	1.38	1.09	0.56	1.28
WA+PBS	2.25	1.98	0.58	0.54	0.53	1.05
WA+Ginaton	1.57	1.1	1.12	1	0.82	2

Ym1

Group	1	2	3	4	5	6
WS+PBS	0.38	1.6	0.79	1.29	1.02	0.93
WS+Ginaton	1.99	0.83	1.35	1.5	1.12	0.56
WA+PBS	1.25	1.87	2.5	1.97	0.73	1.04
WA+Ginaton	2.67	1.07	0.87	1.05	1.45	0.96

IL-10

Group	1	2	3	4	5	6
WS+PBS	0.63	0.85	1.88	0.81	1.13	0.71
WS+Ginaton	1.33	1.01	1.45	0.68	0.5	1.15
WA+PBS	3.51	1.83	0.62	0.86	1.34	1.19
WA+Ginaton	1.59	0.36	1.31	0.83	3.16	1.18

E WB

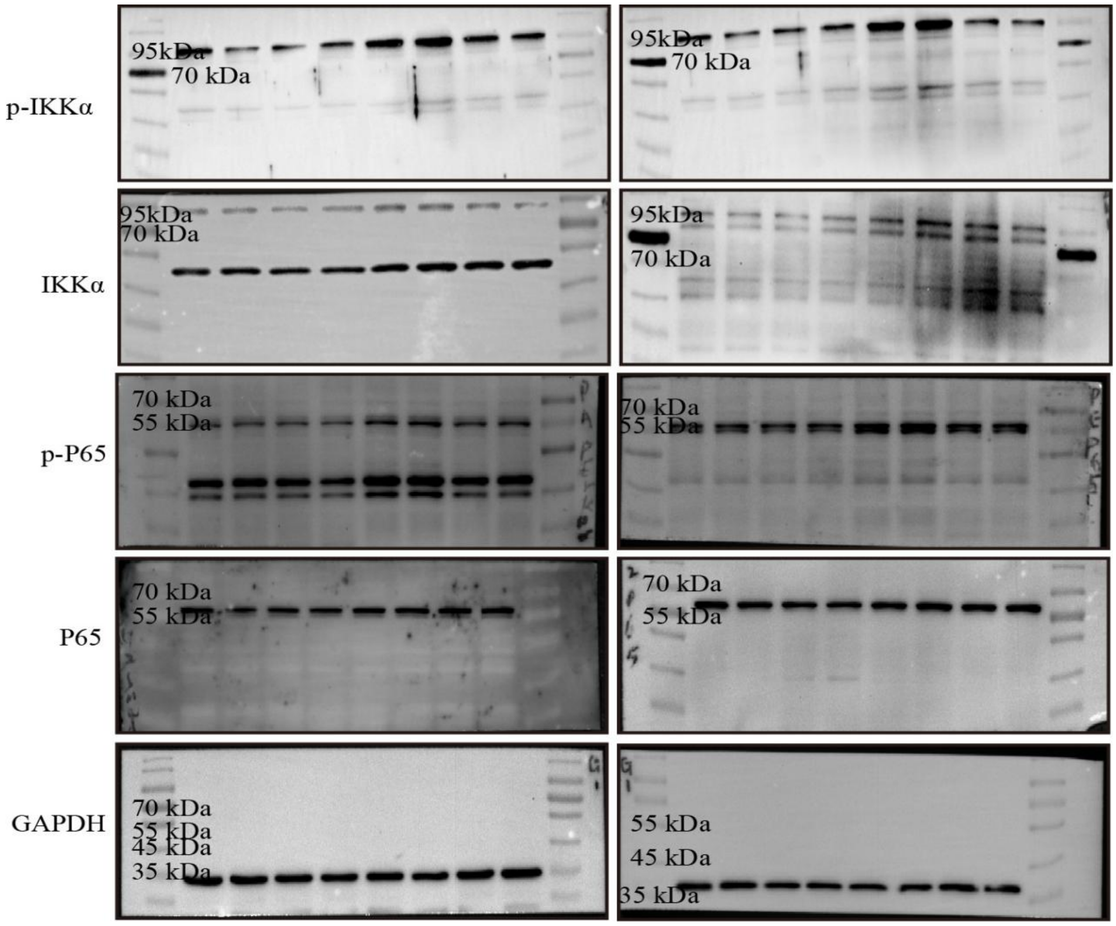

p-IKKα/IKKα

Group	1	2	3	4
WS+PBS	1.62	0.61	1.05	0.7
WS+Ginaton	0.67	1	0.98	1.23
WA+PBS	1.98	2.45	2.34	2.57
WA+Ginaton	1.48	1.57	1.17	0.91

p-P65/P65

Group	1	2	3	4
WS+PBS	0.89	1.12	0.87	1.13
WS+Ginaton	1.03	0.79	1.18	1.11
WA+PBS	1.46	1.73	1.87	1.93
WA+Ginaton	1.12	1.32	1.25	1.12

Figure 5

A iNOS

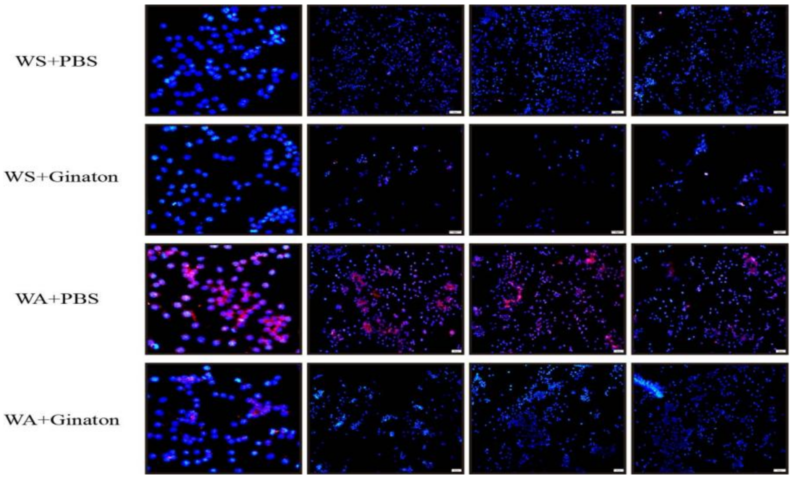

B qPCR

IL-1β

Group	1	2	3
WS+PBS	0.87	1.14	1.02
WS+Ginaton	1.24	0.89	0.76
WA+PBS	3.34	2.72	2.03
WA+Ginaton	1.37	1.65	0.9

IL-6

Group	1	2	3
WS+PBS	0.72	1.4	0.89
WS+Ginaton	0.89	0.85	1.46
WA+PBS	2.45	2.53	1.96
WA+Ginaton	1.62	0.94	1.05

TNF-α

Group	1	2	3
WS+PBS	0.82	1.22	0.97
WS+Ginaton	0.88	0.77	1.01
WA+PBS	2.08	2.34	3.33
WA+Ginaton	1.28	0.85	1.13

MCP-1

Group	1	2	3
WS+PBS	0.98	1.04	0.99
WS+Ginaton	1.24	0.99	1.1
WA+PBS	2.82	2.94	2.04
WA+Ginaton	0.95	1.2	0.9

C CD206

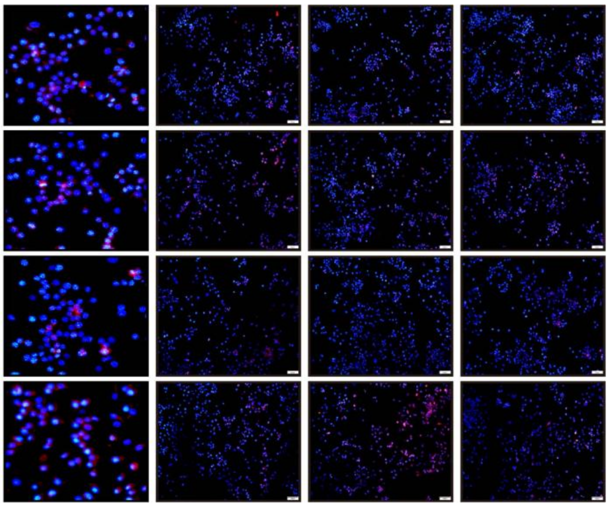

D qPCR

Arg1

Group	1	2	3
WS+PBS	0.34	0.76	1.9
WS+Ginaton	0.55	1.1	1.33
WA+PBS	0.98	1.15	1.01
WA+Ginaton	0.9	0.52	0.51

Ym1

Group	1	2	3
WS+PBS	1.04	0.77	1.19
WS+Ginaton	1.12	1.26	1.96
WA+PBS	1.99	1.6	1.74
WA+Ginaton	1.4	0.92	0.87

IL-10

Group	1	2	3
WS+PBS	0.13	1.55	1.32
WS+Ginaton	1.82	1.15	0.79
WA+PBS	1.86	1.43	1.31
WA+Ginaton	1.41	1.67	0.98

Figure 6

A Adhesion

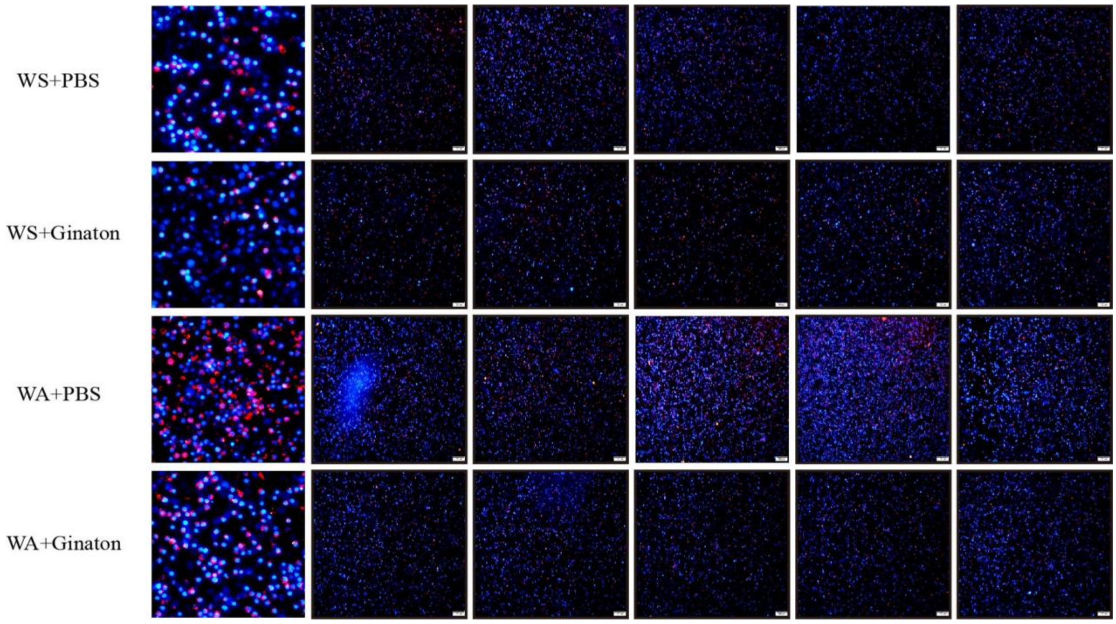

Group	1	2	3	4	5	6
WS+PBS	44	28	35	45	49	40
WS+Ginaton	33	30	26	45	50	49
WA+PBS	162	118	182	100	155	147
WA+Ginaton	100	89	50	56	77	71

B Migration

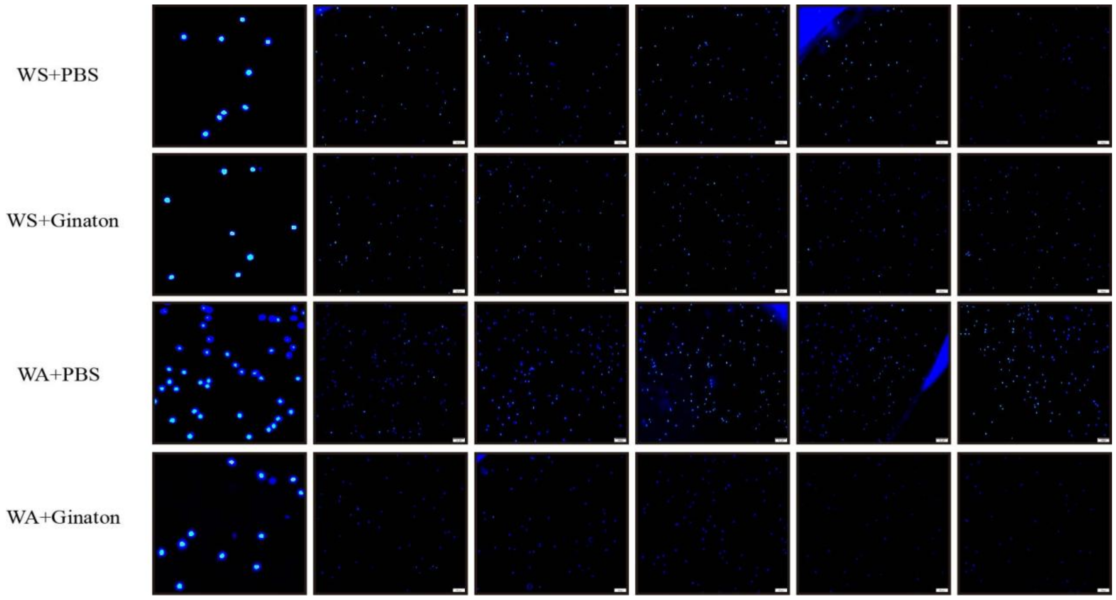

Group	1	2	3	4	5	6
WS+PBS	58	46	54	60	81	57
WS+Ginaton	67	61	86	49	46	51
WA+PBS	217	220	140	127	142	153
WA+Ginaton	77	56	83	40	69	97
